# Hesperetin, a Selective Phosphodiesterase 4 Inhibitor, Effectively Suppresses Ovalbumin-Induced Airway Hyperresponsiveness without Influencing Xylazine/Ketamine-Induced Anesthesia

**DOI:** 10.1155/2012/472897

**Published:** 2012-02-12

**Authors:** Chung-Hung Shih, Ling-Hung Lin, Hsin-Te Hsu, Kuo-Hsien Wang, Chi-Yin Lai, Chien-Ming Chen, Wun-Chang Ko

**Affiliations:** ^1^Department of Internal Medicine, Taipei Medical University Hospital, Taipei 110, Taiwan; ^2^Department of Dentistry, Taipei Medical University Hospital, Taipei 110, Taiwan; ^3^Department of Otolaryngology, Taipei Medical University Hospital, Taipei 110, Taiwan; ^4^Department of Dermatology, Taipei Medical University Hospital, Taipei 110, Taiwan; ^5^Department of Pharmacology, College of Medicine, Taipei Medical University, 250 Wu-Hsing Street, Taipei 110, Taiwan; ^6^Department of Medical Technology, College of Medicine, Taipei Medical University, Taipei 110, Taiwan

## Abstract

Hesperetin, a selective phosphodiesterase (PDE)4 inhibitor, is present in the traditional Chinese medicine, “Chen Pi.” Therefore, we were interested in investigating its effects on ovalbumin- (OVA-) induced airway hyperresponsiveness, and clarifying its rationale for ameliorating asthma and chronic obstructive pulmonary disease (COPD). Hesperetin was revealed to have a therapeutic (PDE4_H_/PDE4_L_) ratio of >11. Hesperetin (10 ~ 30 *μ*mol/kg, intraperitoneally (i.p.)) dose-dependently and significantly attenuated the airway hyperresponsiveness induced by methacholine. It also significantly suppressed the increases in total inflammatory cells, macrophages, lymphocytes, neutrophils, and eosinophils, and levels of cytokines, including interleukin (IL)-2, IL-4, IL-5, interferon-*γ*, and tumor necrosis factor-*α* in bronchoalveolar lavage fluid (BALF). It dose-dependently and significantly suppressed total and OVA-specific immunoglobulin E levels in the BALF and serum. However, hesperetin did not influence xylazine/ketamine-induced anesthesia, suggesting that hesperetin has few or no emetic effects. In conclusion, the rationales for ameliorating allergic asthma and COPD by hesperetin are anti-inflammation, immunoregulation, and bronchodilation.

## 1. Introduction

Phosphodiesterases (PDEs) are classified according to their primary protein and complementary (c)DNA sequences, cofactors, substrate specificities, and pharmacological roles. It is now known that PDEs comprise at least 11 distinct enzyme families that hydrolyze adenosine 3′,5′ cyclic monophosphate (cAMP) and/or guanosine 3′,5′ cyclic monophosphate (cGMP) [1]. PDE1 ~ 5 isozymes, which are calcium/calmodulin dependent (PDE1), cGMP stimulated (PDE2), cGMP inhibited (PDE3), cAMP specific (PDE4), and cGMP specific (PDE5), were found to be present in the canine trachea [2], guinea pig lungs [3], and human bronchi [4]. PDE3 and PDE4 were identified in the guinea pig airway [5], but other isozymes might also be present. PDE4 may adopt two different conformations which have high (PDE4_H_) and low (PDE4_L_) affinities for rolipram, respectively. In general, it is believed that inhibition of PDE4_H_  is associated with adverse responses, such as nausea, vomiting, and gastric hypersecretion, while inhibition of PDE4_L_ is associated with anti-inflammatory and bronchodilating effects. Therefore the therapeutic ratio of selective PDE4 inhibitors for use in treating asthma and chronic obstructive pulmonary disease (COPD) is defined as the PDE4_H_/PDE4_L_ ratio [6, 7].

Hesperetin (5,7,3′-trihydroxy-4′-methoxyflavanone, mol wt., 302.28), one of the most common flavonoids in *Citrus*, is also present in herbal medicine as glycosides. For example, hesperidin and neohesperidin are abundantly present in the fruit peel of *Citrus aurantium* L. (Rutaceae), a well-known traditional Chinese medicine (TCM) called “Chen-Pi”; they are used as an expectorant and stomach tonic, and contain vitamin P, a remedy for preventing capillary fragility and hypertension [8]. These glycosides are easily hydrolyzed by glycosidase to form hesperetin after ingestion. Men with higher hesperetin intake have lower mortality from cerebrovascular disease and lung cancer, and lower incidences of asthma [9]. Because hesperetin was reported to selectively inhibit PDE4 activity [10], and to inhibit the maturation and function of monocyte-derived dendritic cells from patients with asthma [11]. Therefore, we were interested in investigating the PDE4_H_/PDE4_L_ ratio and suppressive effects of hesperetin on ovalbumin- (OVA-) induced airway hyperresponsiveness (AHR), and clarifying its rationale for ameliorating asthma and COPD.

## 2. Materials and Methods

### 2.1. Reagents and Animals

Hesperetin, OVA, methacholine (MCh), aluminum sulfate hexadecahydrate, dimethylsulfoxide (DMSO), chloralose, urethane, Tris-HCl, Bis-Tris, benzamidine, phenylmethanesulfonyl fluoride (PMSF), *d,l*-dithiothreitol, polyethyleneimine, ethylenediaminetetraacetic acid (EDTA), bovine serum albumin (BSA), cAMP, cGMP, calmodulin, Dowex resin, *Crotalus atrox *snake venom, xylazine, and ketamine were purchased from Sigma Chemical (St. Louis, MO, USA). Vinpocetine, *erythro*-9-(2-hydroxy-3-nonyl)-adenine HCl (EHNA), milrinone, 4-(3-butoxy-4-methoxybenzyl)-2-imidazolidinone (Ro 20-1724), and zaprinast were purchased from Biomol (Plymouth Meeting, PA, USA). Freund's adjuvant (*Mycobacterium butyricum*) was purchased from Pierce Biotechnology (Rockford, IL, USA). Mouse T helper (Th)1/Th2 cytokine CBA kits, and mouse IgE enzyme-linked immunosorbent assay (ELISA) sets were purchased from Pharmingen (San Diego, CA, USA). Ethyl alcohol and polyethylene glycol (PEG) 400 were purchased from Merck (Darmstadt, Germany). [^3^ *H*]-cAMP, [^3^ *H*]-cGMP, and [*methyl-* ^3^ *H*]-rolipram were purchased from Amersham Pharmacia Biotech (Buckinghamshire, UK). Other reagents, such as CaCl_2_, MgCl_2_, and NaCl, were of analytical grade. Hesperetin, rolipram, and Ro 20-1724 were dissolved in a mixture of ethyl alcohol and DMSO (1 : 1). Other reagents were dissolved in distilled water.

Male Hartley guinea pigs (500 ~ 600 g) and female BABL/c mice at 8 ~ 12 weeks old were purchased from the Animal Center of the National Science Council (Taipei, Taiwan), and housed in ordinary cages at 22 ± 1°C with a humidity of 50% ~ 60% under a constant 12/12-h light/dark cycle and provided with food and water *ad libitum*. Under a protocol approved by the Animal Care and Use Committee of Taipei Medical University, the following *in vivo* experiments were performed.

### 2.2. Competitive Inhibition of PDE4 Activity by Hesperetin

Activity of PDE4 in the homogenate of guinea pig lungs or hearts was measured by a two-step procedure according to the previous method [12], using cAMP with [^3^ *H*]-cAMP as substrate. The enzyme preparation (25 *μ*L) was incubated for 30 min at 37°C in a total assay volume of 100 *μ*L containing 50 mM Tris-HCl (pH 7.4), 3 mM MgCl_2_, 1 mM dithiothreitol, 0.05% BSA, and 1 *μ*M cAMP with 0.2 *μ*Ci [^3^ *H*]-cAMP as a substrate alone. In the Lineweaver-Burk analysis, the reaction mixture contained 10 *μ*L of vehicle or inhibitors, at various concentrations of hesperetin or rolipram, a selective PDE4 inhibitor [13] as a reference drug, respectively. The reagents and homogenate were mixed on ice, and the reaction was initiated by transferring the mixture to a water bath at 37°C. Following a 30 min incubation, the reaction was stopped by transferring the reaction vessel to a bath of boiling water for 3 min. After cooling on ice, 20 *μ*L of a 1 mg/mL solution of *Crotalus atrox* snake venom was added to the reaction mixture, and the mixture was incubated at 37°C for 10 min. Unreacted [^3^ *H*]-cAMP was removed by the addition of 500 *μ*L of a 1-in-1 Tris-HCl (40 mM) buffer suspension of Dowex resin (1 × 8-200) with incubation on ice for 30 min. Each tube was then centrifuged at 3700 g for 2 min, and 150 *μ*L of the supernatant was removed for liquid scintillation counting. Less than 10% of the tritiated cyclic nucleotide was hydrolyzed in this assay. The total protein in each fraction used was assayed according to the previous method [14]. PDE activity is reported as nmol/mg/min.

### 2.3. Determination of PDE4_**H**_ Values

When the above-mentioned guinea pigs were sacrificed, the whole brains were removed and homogenized with a glass/Teflon homogenizer (Glas-Col, Terre Haute, IN, USA) in 10 volumes of cold medium (pH 6.5) containing 20 mM Bis-Tris, 2 mM benzamidine, 2 mM EDTA, 50 mM sodium chloride, 0.1 mM PMSF, and 1 mM dithiothreitol. At 4°C, the homogenate was centrifuged at 170 g for 5 min to remove connective tissues and blood vessels. The suspended homogenate was then recentrifuged at 40,000 g for 30 min to separate the cytosolic and particulate portions. The particulate portion was resuspended in a suspension at a concentration of 400 mg/mL (wet weight/volume), after washing 3 times with homogenizing buffer. The particulate portion mainly consisted of cell membranes. The binding ability of hesperetin (3 ~ 300 *μ*M) on high-affinity rolipram-binding sites (HARBSs) of membranes was determined by replacing 2 nM [^3^ *H*]-rolipram in a reaction buffer at 30°C for 1 h, according to the method described by previous investigators [13, 15] and modified by us. Briefly, the reaction buffer consisted of 50 mM Tris-HCl and 5 mM MgCl_2_ (pH 7.5). The total volume of the reaction mixture was 25 *μ*L, consisting of 10 *μ*L of the particulate suspension, 10 *μ*L of [^3^ *H*]-rolipram, and 5 *μ*L of hesperetin or reference drugs, such as rolipram (0.1 ~ 1,000 nM) and Ro 20-1724 (1 ~ 10,000 nM) [16]. After 1 h, the reaction was terminated by moving the reaction vessel into crushed ice. Then the reaction mixture was transferred onto Whatman GF/B glass-fiber filters, which were soaked in a 0.3% polyethyleneimine solution in a minifunnel. The reaction mixture was filtered by centrifugation at 90 g for 10 s, and the filtrate was collected into a 1.5 mL Eppendorf tube with the top adapted to the outlet of the minifunnel. The filters were washed with 300 *μ*L of reaction buffer three times each in the same way, and transferred into 2 mL of cocktail for radiation counting (total binding) using a *β*-scintillation counter (Beckman, Fullerton, CA, USA). Nonspecific binding, which was defined in the presence of 10 *μ*M Ro 20-1724, was subtracted from total binding to yield specific binding. Effective concentration (EC_50_) values of hesperetin, rolipram, and Ro 20-1724, at which a half of the [^3^ *H*]-rolipram that was bound onto HARBSs of cell membranes was displaced, were defined as PDE4_H_ values, and these were related to any adverse effects, such as nausea, vomiting, and hypergastric secretion [7].

### 2.4. Airway Hyperresponsiveness (AHR)

*In vivo*, ten female BABL/c mice in each group were sensitized by an intraperitoneal (i.p.) injection of 20 *μ*g of OVA emulsified in 2.25 mg of an aluminum hydroxide gel, prepared from aluminum sulfate hexadecahydrate, in a total volume of 100 *μ*L on days 0 and 14. On day 21, these mice were injected with (i.p.) 100 *μ*L of a mixture of 1% OVA and Freund's complete adjuvant (1 : 1). Mice were challenged *via* the airway using 1% OVA in saline for 30 min on days 28, 29, and 30 by ultrasonic nebulization. After the last of the primary OVA challenges [17], AHR was assessed on day 32 (48 h after 1% OVA provocation) in each group. Each group of mice was administered (i.p.) the vehicle (control) or 3 ~ 30 *μ*mol/kg of hesperetin 2 h before and 6 and 24 h after OVA provocation. For comparison, sham-treated mice were sensitized but challenged with saline instead of 1% OVA (nonchallenged). The vehicle, a mixture of DMSO : ethyl alcohol : PEG 400 : saline (0.5 : 0.5 : 14.5 : 14.5, v/v), or hesperetin was administered (i.p.) at a volume of 0.01 mL/g of body weight. AHR was assessed in unrestrained animals by barometric plethysmography [18] using a whole-body plethysmograph (WBP) and analyzed using software of Life Science Suite P3 Analysis Modules (Gould, LDS Test and Measurement LLC, Valley View, OH, USA). Mice were placed into the main chamber of the WBP, and the baseline enhanced pause (*P*_enh_) value was determined. Then mice were first nebulized with phosphate-buffered saline (PBS), and subsequently with increasing doses (6.25 ~ 50 mg/mL) of MCh for 3 min for each nebulization, followed by readings of breathing parameters for 3 min after each nebulization to determine *P*_enh_ values. Twenty-four hours after *P*_enh_ determination, these mice were anesthetized with pentobarbital (50 mg/kg, i.p.), and the lungs were lavaged *via* a tracheal tube with PBS (1 × 1.0 mL, 37°C). After lavage, blood was collected from the jugular vein and allowed to sit so that it would coagulate. The collected bronchoalveolar lavage fluid (BALF) and coagulated blood were, respectively, centrifuged at 630 g for 7 min and at 3700 g for 10 min at 4°C. After centrifugation, the BALF and serum supernatants were stored at −20°C until determination of cytokines, including interleukin (IL)-2, IL-4, IL-5, tumor necrosis factor (TNF)-*α*, and interferon (IFN)-*γ* by flow cytometric methods [19] using mouse T helper (Th)1/Th2 cytokine CBA kits, and of total immunoglobulin (Ig)E using ELISA kits (Pharmingen, San Diego, CA, USA) according to the respective recommendations of the manufacturers. OVA-specific IgE was measured as described previously [20]. Wells were coated with 100 *μ*L of OVA (20 *μ*g/mL) instead of the capture antibody. Levels are expressed in arbitrary units, where 1 arbitrary unit equals the optical density of the sample divided by the optical density of unchallenged mouse serum or BALF (standard). The BALF pellet was resuspended in ACK lysing buffer (1.658 g NH_4_Cl, 0.2 g KHCO_3_, and 1.44 mg EDTA in 200 mL of water) to lyse the residual erythrocytes in each sample. The number of inflammatory cells was counted using a hemocytometer (Hausser Scientific, Horsham, PA, USA). Cytospun slides were stained and differentiated in a blinded fashion by counting at least 100 cells under light microscopy.

### 2.5. Xylazine/Ketamine-Induced Anesthesia

According to a previously described method [21] and modified by us, hesperetin (10 ~ 100 *μ*mol/kg, subcutaneously (s.c.)) or Ro 20-1724  (0.01 ~ 1 *μ*mol/kg, s.c.), a reference drug, was, respectively, injected into 8 ~ 12-week-old female BALB/c mice 1 or 0.25 h prior to an i.p. injection of xylazine (10 mg/kg)/ketamine (70 mg/kg). The vehicle (control) for hesperetin or Ro 20-1724 was a mixture of DMSO : ethyl alcohol : PEG 400 : saline (0.5 : 0.5 : 14.5 : 14.5, v/v). After loss of the righting reflex (i.e., when a mouse remained on its back and no longer spontaneously righted itself to a prone position), the duration of anesthesia was measured until its return as the endpoint [21].

### 2.6. Statistical Analysis

All values are given as the mean ± SEM. Differences among values were statistically calculated by one-way analysis of variance (ANOVA), and then determined by Dunnett's test. The difference between two values, however, was determined by Student's *t*-test. Differences with *P* < 0.05 were considered statistically significant.

## 3. Results

### 3.1. Competitive Inhibition of PDE4 Activity by Hesperetin

**Figure 1:**
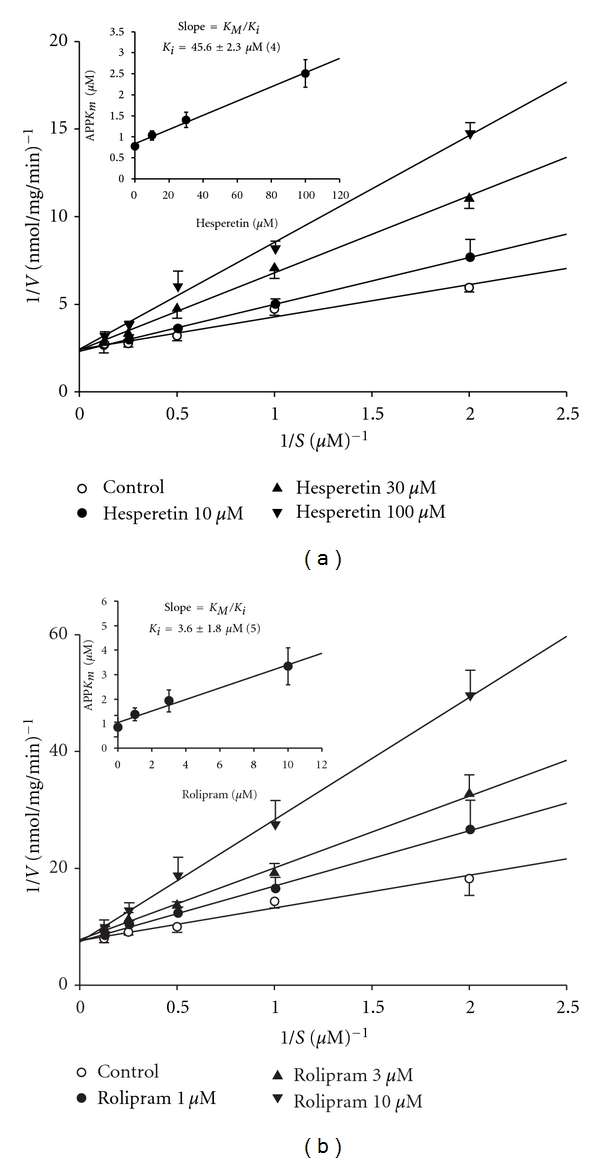
Inhibition of PDE4 induced cAMP hydrolysis by hesperetin (a) and rolipram (b). Activities of PDE4 in the presence of various concentrations of hesperetin or rolipram, and the substrate (cAMP) were plotted according to a Lineweaver-Burk analysis. *K*_*i*_ was determined from the equation of the apparent *K*_*m*_ as a function of the inhibitor concentration (inset). Each value represents the mean ± SEM. The experimental number for hesperetin, and rolipram was 4 and 5, respectively.

According to the Lineweaver-Burk analysis, hesperetin (10 ~ 100 *μ*M) and rolipram (1 ~ 10 *μ*M) competitively inhibited PDE4 activity (Figure 1), because 1/V_max_ values were not significantly affected by various concentrations of hesperetin (a) or rolipram (b). Their *K*_*i*_ values were, respectively, calculated to be 45.6 ± 2.3 (*n* = 4) and 3.6 ± 1.8 (*n* = 5) *μ*M (Figure 1 inset).

### 3.2. PDE4_**H**_ Values

**Figure 2:**
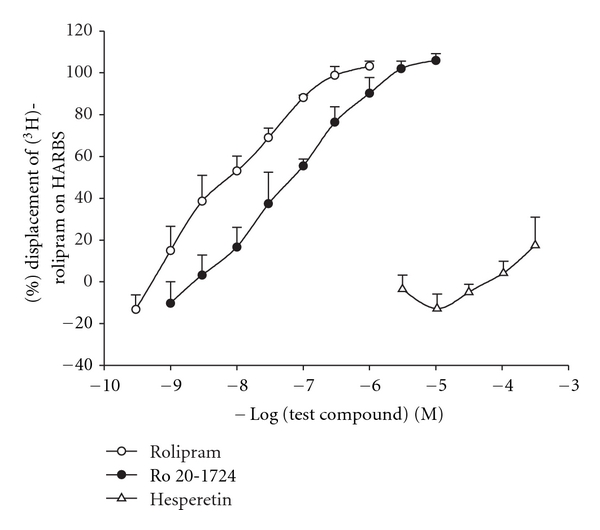
Displacement of [^3^H]-rolipram by rolipram, Ro 20-1724, and hesperetin in high-affinity rolipram binding sites of guinea pig brain particulate. Each value represents the mean ± SEM. The experimental number for each was 4.

Rolipram (0.1 ~ 1,000 nM) and Ro 20-1724 (1 ~ 10,000 nM), concentration-dependently and effectively displaced 2 nM [^3^H]-rolipram binding on HARBSs of guinea pig brain cell membranes (Figure 2). However, hesperetin even at 300 *μ*M displaced those only by 17.5 ± 9.5% (*n* = 4) (Figure 2). The respective EC_50_ (PDE4_H_) values of rolipram, Ro 20-1724, and hesperetin for displacing [^3^H]-rolipram binding were 7.5 ± 3.4 (*n* = 4) nM, 45.6 ± 9.7 (*n* = 4) nM, and >300 *μ*M.

### 3.3. Supsression of Airway Hyperresponsiveness *In Vivo*

**Figure 3:**
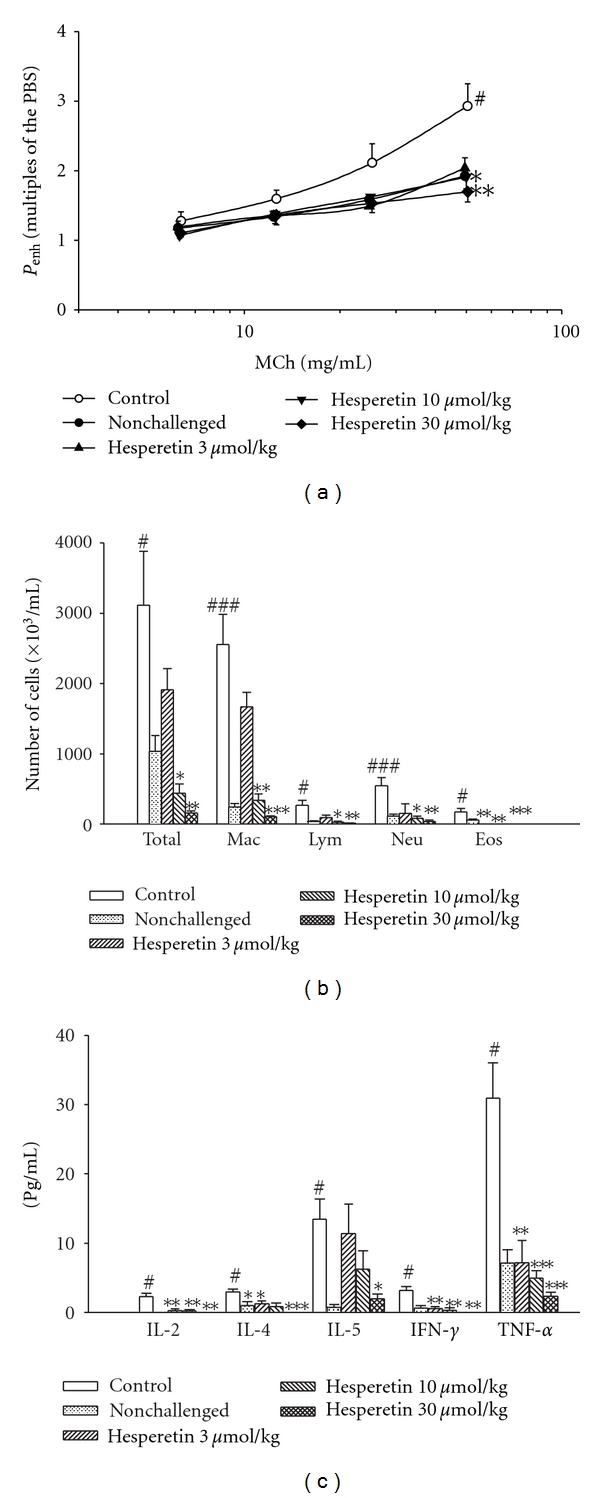
Effects of hesperetin (10 ~ 100 *μ*mol/kg, i.p.) on the enhanced pause (*P*_enh_) (a), inflammatory cells (b), and cytokines (c) in sensitized mice which received aerosolized methacholine (6.25 ~ 50 mg/mL) 2 days after primary allergen challenge. ^#^ *P* < 0.05, ^##^ *P* < 0.01, and ^###^ *P* < 0.001, compared to the nonchallenged group. *P < 0.05, **P < 0.01, and ***P < 0.001, compared to the control (vehicle) group. The number of mice in each group was 10. Total: total cells; Mac: macrophages; Lym: lymphocytes; Neu: neutrophils; Eos: eosinophils; IL: interleukin; IFN: interferon; TNF: tumor necrosis factor.

*P*_ enh_ values at the baseline for the control (vehicle), nonchallenged, and 3, 10, and 30 *μ*mol/kg hesperetin groups were 2.37 ± 0.04, 2.40 ± 0.05, 2.42 ± 0.03, 2.35 ± 0.05, and 2.42 ± 0.04, respectively, and these values did not significantly differ from each other. *P*_enh_ values with PBS nebulization for each group were 2.42 ± 0.05, 2.43 ± 0.03, 2.38 ± 0.05, 2.36 ± 0.04, and 2.44 ± 0.05, respectively, which also did not significantly differ from each other. Administration of nebulized PBS did not affect the *P*_enh_ value of the baseline in each group. However, MCh (6.25 ~ 50 mg/mL) concentration-dependently increased *P*_enh_ values from 1-fold with PBS exposure to 2.93 ± 0.32-fold in control sensitized and challenged mice (Figure 3(a)). *P*_enh_ values of MCh at 50 mg/mL in control sensitized and challenged mice were significantly enhanced compared to those in non-challenged mice. Hesperetin (10 ~ 30  *μ*mol/kg, i.p.) dose-dependently and significantly attenuated the enhancement of *P*_enh_ values induced by 50 mg/mL MCh (Figure 3(a)).

### 3.4. Suppression of Inflammatory Cells in BALF

Total inflammatory cells, macrophages, lymphocytes, neutrophils, and eosinophils from the BALF of control sensitized and challenged mice significantly increased compared to those of nonchallenged mice (Figure 3(b)). Hesperetin (10 ~ 30 *μ*mol/kg, i.p.) significantly suppressed the increases in total inflammatory cells, macrophages, lymphocytes, neutrophils, and eosinophils. Hesperetin even at 3 *μ*mol/kg (i.p.) also suppressed the increase of eosinophils (Figure 3(b)).

### 3.5. Suppression of Cytokines in BALF

Compared to those in nonchallenged mice, levels of cytokines, such as IL-2, IL-4, IL-5, IFN-*γ*, and TNF-*α*, in the BALF of control sensitized and challenged mice significantly increased (Figure 3(c)). Hesperetin (3 ~ 30 *μ*mol/kg, i.p.) significantly suppressed increases in levels of IL-2, IL-4, IL-5, IFN-*γ*, and TNF-*α* with the exception of IL-5 at doses of 3 and 10 *μ*mol/kg (Figure 3(c)).

### 3.6. Suppression of IgE in the Serum and BALF

**Figure 4:**
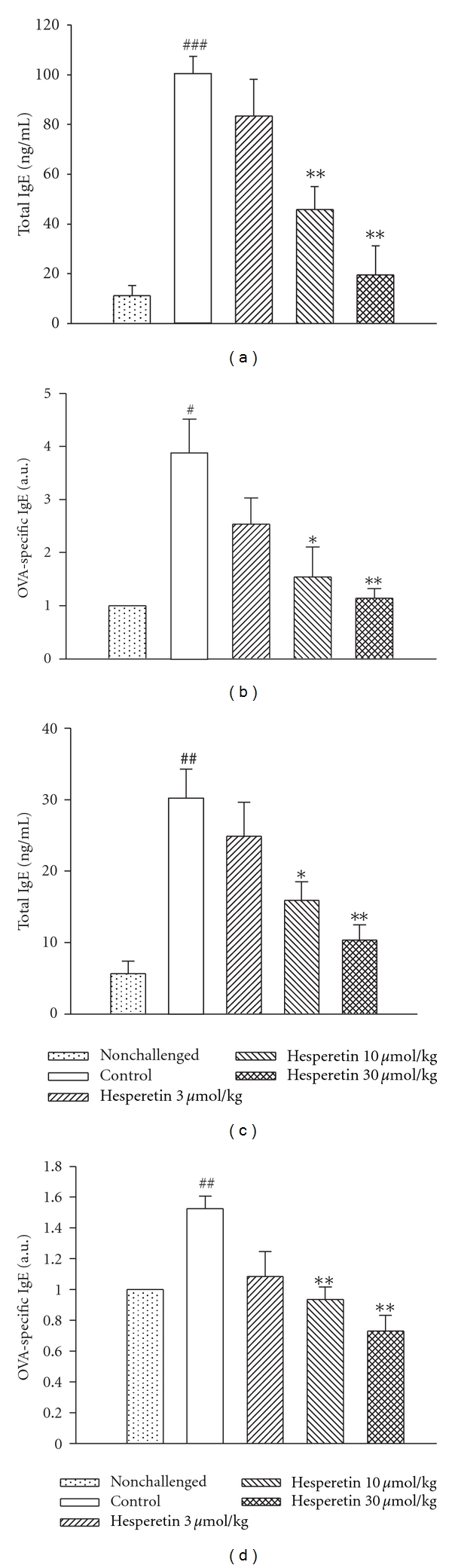
Effects of hesperetin (3 ~ 30 *μ*mol/kg, i.p.) on total IgE (a, c) and ovalbumin-specific IgE (b, d) levels in bronchial alveolar lavage fluid (a, b) and serum (c, d) of sensitized mice which had received aerosolized methacholine (6.25 ~ 50 mg/mL) 2 days after primary allergen challenge. ^#^ *P* < 0.05, ^##^ *P* < 0.01 and ^###^ *P* < 0.001, compared to the nonchallenged group. *P < 0.05, **P < 0.01 and ***P < 0.001, compared to the control (vehicle) group. Each value represents the mean ± SEM. The number of mice in each group was 10.

Levels of total and OVA-specific IgE in the BALF and serum of control sensitized and challenged mice were significantly enhanced compared to those of non-challenged mice. For example, Hesperetin (10 ~ 30 *μ*mol/kg, i.p.) dose-dependently and significantly suppressed these enhancements (Figures 4(a), 4(b), 4(c), and 4(d)).

### 3.7. No Effect on Xylazine/Ketamine-Induced Anesthesia

**Figure 5:**
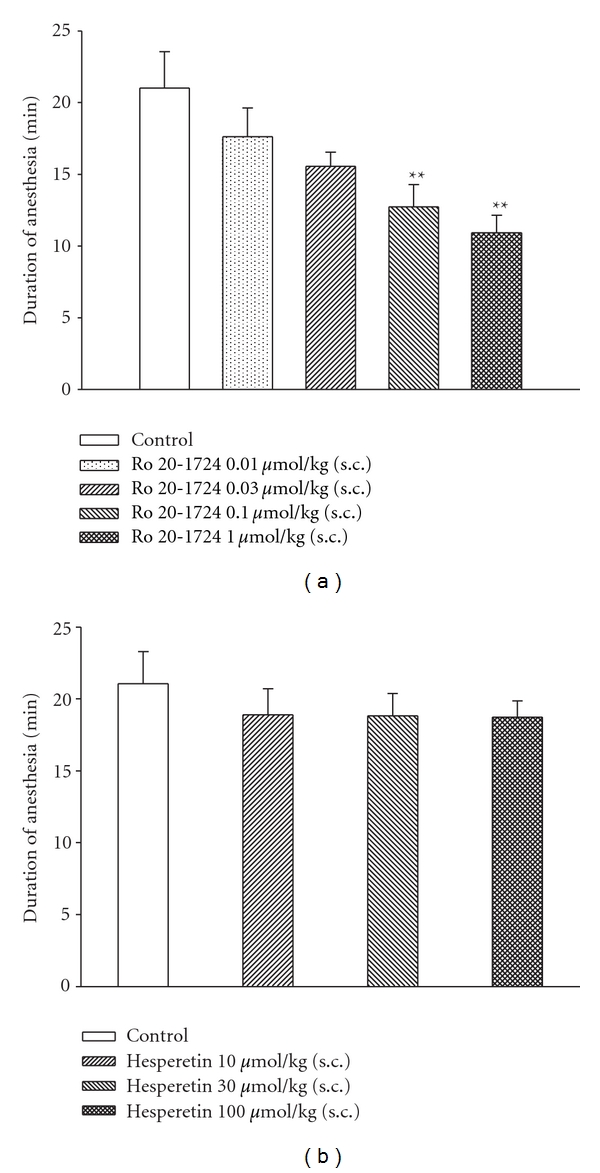
Effects of subcutaneously administered Ro 20-1724 (a) and hesperetin (b) on the duration of xylazine (10 mg/kg, i.p.)/ketamine-induced (70 mg/kg, i.p.) anesthesia in mice. Ro 20-1724 was administered 0.25 h and hesperetin 1 h before anesthesia. **P < 0.01, compared to the vehicle (control). Each value represents the mean ±  SEM. The number of mice in each group was 10.

The durations of xylazine/ketamine-induced anesthesia in control (vehicle) mice of the Ro 20-1724- and hesperetin-treated groups were 21.0 ± 2.5 (*n* = 10) and 22.1 ± 2.2 min (*n* = 10), respectively. Ro 20-1724 (0.01 ~ 1 *μ*mol/kg, s.c.) dose-dependently shortened the duration, and at doses of 0.1 and 1 *μ*mol/kg (s.c.) significantly shortened the duration (Figure 5(a)). In contrast, hesperetin (10 ~ 100 *μ*mol/kg, s.c.) did not significantly influence the duration (Figure 5(b)).

## 4. Discussion

Allergic asthma is a chronic respiratory disease characterized by AHR, mucus hypersecretion, bronchial inflammation, and elevated IgE levels. Th2 cells, together with other inflammatory cells such as eosinophils, B cells, and mast cells were proposed as critical to the initiation, development, and chronicity of this disease [22]. One hypothesis emphasizes an imbalance in Th cell populations favoring expression of Th2 over Th1 cells. Cytokines released from Th2 cells are IL-4, IL-5, IL-6, IL-9, and IL-13, and those from Th1 cells are IL-2, IL-12, IFN-*γ*, and TNF-*α* [23, 24]. In the present results, hesperetin (10 ~ 30 *μ*mol/kg, i.p.) significantly reduced *P*_enh_  values at 50 mg/mL MCh (Figure 3(a)) suggesting that it significantly suppresses AHR. All types of inflammatory cells examined, including total inflammatory cells, macrophages, lymphocytes, neutrophils, and eosinophils in the BALF of sensitized and challenged mice were reduced (Figure 3(b)). Hesperetin (10 ~ 30 *μ*mol/kg, i.p.) also suppressed levels of IL-2, IL-4, IL-5, IFN-*γ*, and TNF-*α* (Figure 3(c)). These results suggest that hesperetin suppresses both Th1 and Th2 cells which are respectively implicated in autoimmune and atopic diseases [25].

IL-4 and IL-13 were shown to induce AHR in mouse asthma models [26, 27]. IL-4 has three primary effects. First, IL-4 promotes B cell differentiation to plasma cells that secrete antigen-specific IgE antibodies. Second, IL-4 promotes mast cell proliferation. Third, increased IL-4 upregulates endothelial cell expression of adhesion molecules for eosinophils [28]. IL-5 mobilizes and activates eosinophils, leading to the release of a major basic protein, cysteinyl-leukotriene, and eosinophil peroxidase that contribute to tissue damage and AHR [27, 29]. Phosphoinositide 3-kinase *δ* (p110*δ*) was shown to play a crucial role in the development, differentiation, and antigen receptor-induced proliferation of mature B cells [30, 31], and inhibition of p110*δ* attenuates allergic airway inflammation and AHR in a murine asthma model [30, 32]. In addition, IL-4 and IL-13 are important in directing B cell growth, differentiation, and secretion of IgE [33]. In addition, hesperetin (10 ~ 30 *μ*mol/kg, i.p.) dose-dependently and significantly suppressed total and OVA-specific IgE levels in the BALF and serum of sensitized and challenged mice, suggesting that hesperetin has immunoregulatory and antiallergic asthmatic effects. The results support the recent finding that orally administered hesperidin (hesperetin-7-rutinoside or hesperetin-7-rhamnoglucoside), inhibited inflammatory cell infiltration and mucus hypersecretion in a murine model of asthma [34].

Hesperetin has been reported to selectively inhibit PDE4 activity in our previous report [10], and in the present results, it was revealed to competitively inhibit PDE4 activity. Selective PDE4 inhibitors specifically prevent the hydrolysis of cAMP, a 3′,5′-cyclic nucleotide, and therefore have broad anti-inflammatory effects such as inhibition of cell trafficking and of cytokine and chemokine release from inflammatory cells. The increased cAMP levels induced by these selective PDE4 inhibitors subsequently activate cAMP-dependent protein kinase which may phosphorylate and inhibit myosin light-chain kinase, thus inhibiting contractions [35]. The precise mechanism through which relaxation is produced by this second-messenger pathway is not known, but it may result from decreased intracellular Ca^2+^ ([Ca^2+^]_i_). The decrease in [Ca^2+^]_i_ may be due to reduced influx of Ca^2+^, enhanced Ca^2+^ uptake into the sarcoplasmic reticula, or enhanced Ca^2+^ extrusion through cell membranes [35]. Thus selective PDE4 inhibitors may have bronchodilatory effects. The second-generation PDE4 inhibitors, cilomilast and roflumilast, have reached the clinical trial stage and exhibit some beneficial effects in treating asthma and COPD [36]. The effectiveness of these PDE4 inhibitors may be limited by their clinical potency when using doses that have minimal adverse effects such as headaches, diarrhea, nausea, and abdominal pain. The PDE4_H_/PDE4_L_ ratios of cilomilast and roflumilast were, respectively, reported to be 117.8 nM/120 nM (1), and 2.4 nM/0.8 nM (3) [15, 37], which are considerably greater than that (0.01 ~ 0.001) of rolipram [7]. Owing to its adverse effects or lack of efficacy, cilomilast was discontinued for use against asthma after phase II clinical trials in 2003 [36]. In terms of tolerability over 6 months with 15 mg twice daily for COPD in a phase III study, cilomilast was reported to be associated with higher frequencies of diarrhea and nausea than a placebo [36]. Roflumilast was evaluated for asthma and COPD in phase III clinical trials, and was reported to reduce those adverse effects after longer-term treatment at 0.5 mg once daily [36]. Roflumilast, compared to a placebo, was reported to significantly improve the mean pre- and postbronchodilator forced expiratory volumes in 1 s (FEV_1_) in patients with moderate-to-severe COPD. However, nausea, diarrhea, weight loss, and headaches were more frequent in patients in the roflumilast group. These adverse events were associated with increased patient withdrawal [38, 39]. Recently, roflumilast was approved by the European Commission as an add-on to bronchodilator therapy for maintenance treatment of severe COPD associated with chronic bronchitis in adults with a history of frequent exacerbations. However, the US Food and Drug Administration voted against using roflumilast to treat COPD. The PDE4_H_/PDE4_L_ ratio of AWD 12-281, another selective PDE4 inhibitor, was reported to be 104 nM/9.7 nM (approximately 11) [40]. AWD 12-281 was undergoing clinical development phase IIa trials for COPD, and was reported to be a unique potential drug for the topical treatment of asthma and COPD [41]. AWD 12-281 was reported to be a very promising drug candidate for treating lung inflammation when administered by inhalation and for treating atopic dermatitis [42]. However, AWD-12-281 was also discontinued in clinical trials for both asthma and COPD owing to a lack of efficacy [43, 44]. Many compounds that are in development will not reach the market as monotherapies unless their emetic liability is reduced [45], although inhaled GSK256066 demonstrated efficacy in trials in asthma [46] and oral apremilast was clinically reported to be effective for treating severe plaque-type psoriasis [47]. PDE4 subtypes (A ~ D) may be considered for drug development of new PDE4 inhibitors. PDE4D inhibition in nontarget tissues promotes emesis, since PDE4D knock-out mice showed reduction of xylazine/ketamine-triggered anesthesia which is used as a surrogate marker for emesis in mice, a nonvomiting species [21]. Recently, small-molecule allosteric modulators of PDE4D that do not completely inhibit enzymatic activity were reported to reduce emesis and have therapeutic benefits of a brain distribution, for such entities as Alzheimer's disease, Huntington's disease, schizophrenia, and depression [48]. In contrast to PDE4D, selective inhibition of PDE4A and/or PDE4B in proinflammatory and immune cells is believed to evoke the therapeutically desired effects of these drugs [49]. Cilomilast has a higher potency for PDE4D compared to PDE4A and PDE4B, while roflumilast is non-selective for these four PDE4 subtypes with similar degrees of inhibition [50]. There is no literature about AWD 12-281′s inhibition of PDE4 subtypes until now. However, whether hesperetin selectively inhibits the PDE4 subtype also needs to be further investigated.

In the present results, the PDE4_H_/PDE4_L_ ratio of hesperetin was calculated to be >11, which is greater than that of AWD 12-281. In addition, hesperetin did not influence xylazine/ketamine-induced anesthesia. However, Ro 20-1724, a selective PDE4 inhibitor, reversed the anesthesia. The reversing effect may occur through presynaptic *α*_2_-adrenoceptor inhibition [51], because MK-912, an *α*_2_-adrenoceptor antagonist, was reported to reverse xylazine/ketamine-induced anesthesia in rats [52] and trigger vomiting in ferrets [51]. In contrast, clonidine, an *α*_2_-adrenoceptor agonist, prevented emesis induced by PDE4 inhibitors in ferrets [51]. The present results also suggest that hesperetin may have few or no adverse effects, such as nausea, vomiting, and gastric hypersecretion.

**Figure 6:**
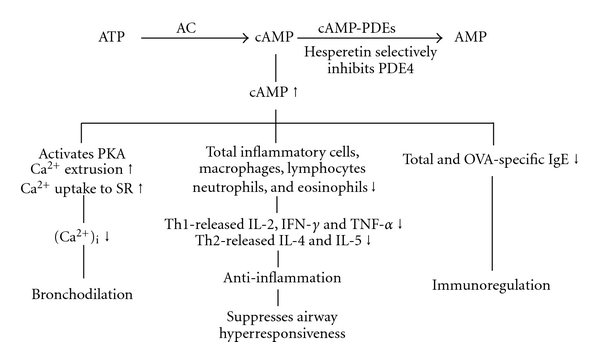
Mechanisms of action of hesperetin. Hesperetin selectively inhibits PDE4 activity and results in an increase in cAMP, which activates cAMP-dependent protein kinase (PKA) and increases calcium extrusion from the intracellular space and uptake to sarcoplasmic reticula (SR). Therefore, hesperetin largely decreases the concentration of intracellular calcium ([Ca^2+^]_i_) and results in bronchodilation. The increase in cAMP also has anti-inflammatory and immunoregulatory effects. AC: adenylate cyclase; Th: T-helper cells; Ig: immunoglobulin; IL: interleukin; IFN: interferon; TNF: tumor necrosis factor. Up and down arrows, respectively, indicate increases and decreases.

In conclusion, hesperetin exerted anti-inflammatory effects, including suppression of AHR, and reduced expressions of inflammatory cells and cytokines in a murine model of allergic asthma. However, hesperetin did not influence xylazine/ketamine-induced anesthesia suggesting that hesperetin has few or no emetic effects. Thus, the rationales for ameliorating allergic asthma and COPD by hesperetin are antiinflammation, immunoregulation, and bronchodilation resulted from PDE4 inhibition and are summarized in Figure 6.

## Abbreviations

AHR: Airway hyperresponsiveness
cAMP: Adenosine 3′,5′ cyclic monophosphate
cGMP: Guanosine 3′,5′ cyclic monophosphate
COPD: Chronic obstructive pulmonary disease
DMSO: Dimethyl sulfoxide
EDTA: Ethylenediaminetetraacetic acid
HARBSs: High-affinity rolipram-binding sites
IFN: Interferon
Ig: Immunoglobulin
IL: Interleukin
*K*_*i*_: Dissociation constant for inhibitor binding
MCh: Methacholine
PBS: Phosphate-buffered saline
PDE: Phosphodiesterase
PDE4_H_: High affinity for PDE4
PDE4_L_: Low affinity for PDE4
*P*_ enh_: Enhanced pause
PMSF: Phenylmethanesulfonyl fluoride
Ro 20-1724: 4-(3-butoxy-4-methoxybenzyl)-2-imidazolidinone
TCM: Traditional Chinese medicine
Th: T-helper
TNF: Tumor necrosis factor.
